# Unsupervised Pattern Analysis to Differentiate Multiple Sclerosis Phenotypes Using Principal Component Analysis on Various MRI Sequences

**DOI:** 10.3390/jcm13175234

**Published:** 2024-09-04

**Authors:** Chris W. J. van der Weijden, Milena S. Pitombeira, Débora E. Peretti, Kenia R. Campanholo, Guilherme D. Kolinger, Carolina M. Rimkus, Carlos Alberto Buchpiguel, Rudi A. J. O. Dierckx, Remco J. Renken, Jan F. Meilof, Erik F. J. de Vries, Daniele de Paula Faria

**Affiliations:** 1 Department of Nuclear Medicine and Molecular Imaging, University of Groningen, University Medical Center Groningen, Hanzeplein 1, 9700 RB Groningen, The Netherlands; c.w.j.van.der.weijden@umcg.nl (C.W.J.v.d.W.);; 2 Department of Radiology, University of Groningen, University Medical Center Groningen, Hanzeplein 1, 9700 RB Groningen, The Netherlands; 3 Laboratory of Nuclear Medicine, Department of Radiology and Oncology, University of Sao Paulo, São Paulo 05508-220, Brazil; 4 Department of Neuroscience, University of Groningen, University Medical Center Groningen, Hanzeplein 1, 9700 RB Groningen, The Netherlands; 5 Department of Biomedical Sciences of Cells and Systems, University of Groningen, University Medical Center Groningen, Hanzeplein 1, 9700 RB Groningen, The Netherlands; 6 Department of Neurology, Martini Ziekenhuis, 9728 NT Groningen, The Netherlands

**Keywords:** differential diagnosis, inhomogeneous magnetisation transfer, multiple sclerosis, precision medicine, scaled subprofile modelling/principal component analysis

## Abstract

**Background**: Multiple sclerosis (MS) has two main phenotypes: relapse-remitting MS (RRMS) and progressive MS (PMS), distinguished by disability profiles and treatment response. Differentiating them using conventional MRI is challenging. **Objective**: This study explores the use of scaled subprofile modelling using principal component analysis (SSM/PCA) on MRI data to distinguish between MS phenotypes. **Methods**: MRI scans were performed on patients with RRMS (n = 30) and patients with PMS (n = 20), using the standard sequences T_1_w, T_2_w, T_2_w-FLAIR, and the myelin-sensitive sequences magnetisation transfer (MT) ratio (MTR), quantitative MT (qMT), inhomogeneous MT ratio (ihMTR), and quantitative inhomogeneous MT (qihMT). **Results**: SSM/PCA analysis of qihMT images best differentiated PMS from RRMS, with the highest specificity (87%) and positive predictive value (PPV) (83%), but a lower sensitivity (67%) and negative predictive value (NPV) (72%). Conversely, T_1_w data analysis showed the highest sensitivity (93%) and NPV (89%), with a lower PPV (67%) and specificity (53%). Phenotype classification agreement between T_1_w and qihMT was observed in 57% of patients. In the subset with concordant classifications, the sensitivity, specificity, PPV, and NPV were 100%, 88%, 90%, and 100%, respectively. **Conclusions**: SSM/PCA on MRI data revealed distinctive patterns for MS phenotypes. Optimal discrimination occurred with qihMT and T_1_w sequences, with qihMT identifying PMS and T_1_w identifying RRMS. When qihMT and T_1_w analyses align, MS phenotype prediction improves.

## 1. Introduction

Multiple sclerosis (MS) is a neurodegenerative disease characterised by inflammatory and demyelinating lesions [1,2]. With an onset of 18–40 years, MS is the most common neurodegenerative disease among young adults with a prevalence of 2.8 million case worldwide [3,4]. Presently, MS is diagnosed through the revised McDonald criteria involving clinical history, neurological examination, and conventional MRI [5]. MS exhibits two main phenotypes: relapsing-remitting MS (RRMS), featuring acute relapses followed by recovery, and progressive MS (PMS), characterised by continuous disability worsening without relapses [6,7]. RRMS is the most common form of MS, accounting for about 85% of initial MS diagnoses [8]. PMS represents a smaller percentage of MS cases at onset (around 10–15% for primary PMS), but many individuals with RRMS may eventually transition to secondary PMS.

Treatment decisions in MS heavily rely on disease activity, which is primarily supported by MRI findings. While RRMS can lead to significant disability over time, the periods of remission provide a chance for recovery, and disease-modifying therapies (DMTs) aimed at reducing the inflammatory response can effectively reduce the frequency and severity of relapses [8]. PMS is generally associated with a more severe prognosis due to the steady accumulation of disability. As inflammation is less pronounced in PMS, DMTs are, in general, less effective in PMS than in RRMS. Managing the PMS population, especially those with initial primary PMS symptoms or transitioning from RRMS to secondary PMS, is challenging due to lower disease activity detected by conventional MRI [9]. Differences in disease characteristics lead to different treatment strategies for RRMS and PMS patients. With emerging therapeutic options for both phenotypes, precise classification of the disease phenotype becomes crucial for optimal use of these often costly treatments [10,11].

The application of advanced MRI sequences could potentially bridge the gap between disability profiles and the underlying pathology, aiding personalised treatment decisions [12,13]. Since MS patients routinely undergo diagnostic MRI scans, a method to differentiate MS phenotypes based on MRI could be easily integrated into routine clinical practice [5]. T_1_w, T_2_w, and T_2_w-FLAIR MRI, known for their high lesion detection sensitivity, are commonly used for MS diagnosis [5,14], whereas magnetisation transfer (MT)-based MRI and diffusion tensor imaging (DTI) are used as research tools to explore myelin sensitivity and neuronal tracts. Yet, qualitative inspection of these images cannot distinguish between the MS phenotypes [15,16].

We hypothesised that statistical methods could reveal nuances in MR images that are challenging to discern qualitatively. Scaled subprofile modelling using principal component analysis (SSM/PCA) [17], an advanced statistical technique, identifies disease patterns by analysing differences between healthy controls (HC) and patients. SSM/PCA has already been successfully applied in neurodegenerative disorders to distinguish different types of Parkinson syndromes [18] and dementias [19]. While predominantly used with PET data, its application to MRI is limited. In this proof-of-concept study, we explored if SSM/PCA can differentiate MS phenotypes using MRI images from routine sequences (T_1_w, T_2_w, T_2_w-FLAIR) or myelin-sensitive MT sequences (qMT, MTR, qihMT, ihMTR) [20].

## 2. Methods

### 2.1. Participants

A total of 35 patients with RRMS and 20 patients with PMS were recruited at the Medical Faculty of the University of São Paulo. All patients were diagnosed with clinically defined MS according to the revised McDonald criteria [14], were relapse free for at least 30 days, and did not have steroid treatment for at least 90 days before MRI acquisition. The exclusion criteria were an age younger than 18, a history of neurological or psychiatric diseases, the presence of metal objects in the body that could not be removed, claustrophobia, pregnancy, or renal, cardiac, or hepatic insufficiency. Disability was assessed using the Expanded Disability Status Scale (EDSS) [21] and clinical phenotypes were determined according to the Lublin classification by a trained neurologist (M.S.P) [6,7]. All participants gave signed informed consent to participate in this prospective study. Two subjects were excluded due to unexpected clinical findings on the MRI, one participant withdrew from the study and did not allow further use of data, one participant was scanned with a different head coil, and one participant received intravenous steroid treatment 5 days before MRI acquisition, which was later reported. This led to a final inclusion of 30 RRMS and 20 PMS patients. The study was conducted according to the Declaration of Helsinki and subsequent revisions and was approved by the medical ethics committee of the University of São Paulo (protocol 3.256.558).

### 2.2. MRI Acquisition

All MRI scans of the brain were performed on a 3T SIGNA PET-MRI scanner (General Electric Company, Waukesha, WI, USA) with a 24-channel head coil. The protocol comprised a 3D-T_1_w (TR/TE/TI = 7.664/3.112/600 ms, voxel size 1 × 0.5 × 0.5 mm), a 3D-T_2_w FLAIR (TR/TE/TI = 6500/141.213/1905 ms, voxel size 1.3 × 0.5 × 0.5 mm), a 3D-T_2_w sequence (TR/TE = 2500/87.494 ms, voxel size 1 × 0.5 × 0.5 mm), and a 3D-inhomogeneous MT sequence (ihMT) (TR/TE = 11.172/2.48 ms, voxel size 2.6 × 1.0 × 1.0 mm).

### 2.3. MRI Post-Processing

The qMT, MTR, qihMT, and ihMTR images were derived from the ihMT sequence [16,20]. The T_2_w, T_2_w-FLAIR, qMT, MTR, qihMT, and ihMTR images were co-registered to the T_1_w image. Segmentation for grey matter (GM), white matter (WM), cerebrospinal fluid (CSF), and lesions was performed using SPM12 (Wellcome Trust Centre for Neuroimaging, London, UK) [22]. All images were then spatially normalised to Montreal Neurological Institute (MNI) space via SPM12 and validated through visual inspection [22]. Whole brain masks, incorporating voxels with GM or WM information across all subjects, were created for SSM/PCA analysis. Global normalisation addressed intensity variations in the T_1_w, T_2_w, and T_2_w-FLAIR images, but was unnecessary for qMT, MTR, qihMT, and ihMTR due to intrinsic normalisation properties. Lesion segmentation for generating differentiating patterns was not performed, as we wanted to identify differentiating brain patterns using information from the whole brain and not exclusively MS lesions.

### 2.4. Statistical Analysis

Statistical analyses were conducted in a blinded setting with coded groups and subjects. Group differences were evaluated using the Kruskal–Wallis or Mann–Whitney U tests in SPSS Statistics, version 23 (IBM, Chicago, IL, USA). For SSM/PCA analysis, a training set comprised 15 RRMS and 15 PMS patients, randomly selected to create distinctive patterns. Automated voxel-based algorithms, described by Spetsiers et al., generated residual profile images by subtracting the RRMS grand mean profile (GMP) from each PMS subject’s scan [23,24]. The GMP is an image containing the average voxel value of 15 RRMS patients. PCA was performed on the residual profile images for each MRI sequence. Components explaining at least 50% of variance were retained for generating PMS-specific patterns. Logistic regression with stepwise forward combination identified PCs making the best group distinction, forming the final phenotype-differentiating pattern. The ‘variance accounted for’ (VAF) quantified the total explained variance. Bootstrap resampling (1000 repetitions) and leave-one-out cross-validation (LOOCV) assessed the pattern stability within each training set. For comprehensive details, refer to Meles et al., 2020 [25].

The subjects that were not used in the training set were used to generate the validation set, which consisted of 15 RRMS and 5 PMS patients. The individual residual profile images were calculated for the subjects of the validation set. The subject score was calculated by projecting the subject’s residual profile onto the disease-specific pattern. If the subject scores in the validation set were significantly higher than the subject scores of RRMS patients in the training set, the subject has a pattern similar to PMS. Receiver operating characteristic (ROC) analysis based on Youden’s method was performed on the LOOCV results to determine a threshold score for the classification of the disease phenotype of the subject. Subsequently, variables like the area under the curve (AUC), specificity, sensitivity, negative predictive value (NPV), positive predictive value (PPV) for differentiation between RRMS and PMS were calculated from the ROC curves.

## 3. Results

### 3.1. Group Characteristics

Demographic characteristics of the included subjects are presented in Table 1. Patients included in the study were off steroid treatment and without major clinical comorbidities. No significant differences in gender or education were found between phenotypes. As expected, the age of PMS patients was significantly higher than that of RRMS patients (H = −4.25, *p* > 0.001). Furthermore, RRMS and PMS patients did not differ in lesion number or volume. The EDSS was significantly higher in PMS patients than in RRMS patients (U = 556, *p* > 0.001), reflecting the difference in the disability profile between the MS phenotypes.

### 3.2. Discriminative Analysis of MRI Patterns for MS Phenotypes

Patterns showing the differences in MRI signal between PMS and RRMS phenotypes were generated, subjected to bootstrapping and LOOCV (Table 2). These patterns could be generated for all MRI sequences, except for MTR, as differences in the MTR signal between phenotypes did not sustain LOOCV, despite the high success rate of bootstrapping (95.5%).

**Figure 1:**
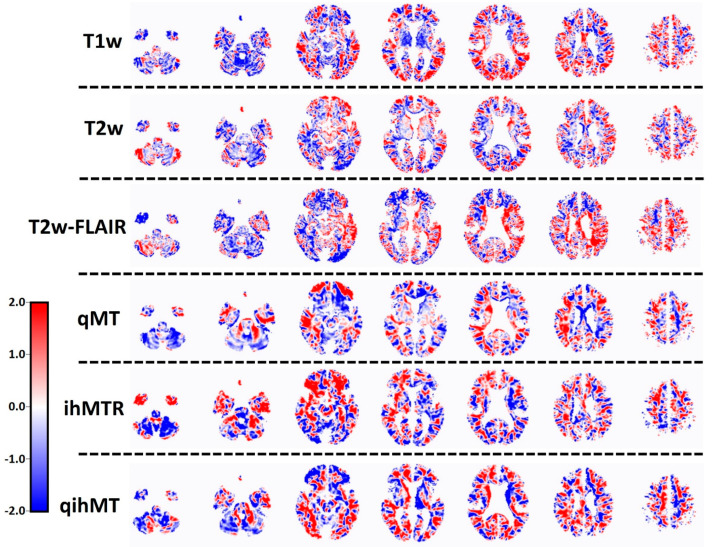
Differentiating brain patterns per MRI method for discriminating RRMS (n = 30) and PMS (n = 20). Rows represent the different MRI methods which could be used to discriminate between MS phenotypes. The columns represent cross-sections of the brain at various depths, ranging from inferior (left) to superior (right). The red colour means that the relative distribution of voxel intensity is increased, and the blue colour means that the relative distribution of voxel intensity is decreased in PMS relative to RRMS. As MRI is measured in arbitrary units, the distribution patterns are also in arbitrary units. T_1_w = T_1_-weighted MRI, T_2_w = T_2_-weighted MRI, T_2_w-FLAIR = T_2_-weighted fluid-attenuated inversion recovery, qMT = quantitative magnetisation transfer, ihMTR = inhomogeneous magnetisation transfer, qihMT = quantitative inhomogeneous magnetisation transfer.

The T_1_w-derived differentiating pattern (Figure 1) shows hypo-intensities in PMS compared to RRMS in the cerebellum and thalamus, whereas hyperintensities were found in the parietal lobe. In addition to that, there seems to be a random allocation of both hypo- and hyper-intensities throughout the brain in the T_1_w differentiating pattern. The T_2_w and T_2_w-FLAIR differentiating patterns reveal hypo-intensities in the cerebellum and occipital lobe of PMS relative to RRMS patients, whereas the peri-ventricular region of PMS is hyper-intense in the T_2_w-FLAIR pattern. In addition, a random distribution of hypo- and hyper-intensities throughout the brain is observed in the T_2_w and T_2_w-FLAIR patterns. qMT and ihMTR also show relative hypo-intensities in the cerebellum of PMS patients, and hyper-intensities in frontal regions. qihMT shows hypo-intensities in the cerebellum and frontal regions and hyper-intensities in the occipital regions of PMS patients as compared to RRMS patients.

qihMT and T_1_w appear to have the highest discriminative power, with the highest AUC (0.68–0.70) in the ROC analysis (Table 3). qihMT has a specificity of 87% and a sensitivity of 67%, whereas T_1_w has two optimal settings, one with a high sensitivity (93%) with a moderate specificity (53%), and one with slightly lower sensitivity (87%) but slightly higher specificity (60%). qihMT has a high positive predictive value (PPV; 83%) and a good negative predictive value (NPV; 72%), whereas T_1_w has a PPV of 68% and 67% and a NPV of 89% and 82% for the two different AUC cut-offs, respectively.

qihMT and T_1_w predicted the same phenotype in 57% and gave discordant results in 43% of the patients. In the concordant group, the combination of qihMT and T_1_w accurately predicted the PMS phenotype with a sensitivity, specificity, PPV, and NPV of 100%, 88%, 90%, and 100%, respectively.

## 4. Discussion

This study assessed the proof-of-concept of MRI to differentiate MS phenotypes using SSM/PCA. Among the different MRI sequences, SSM/PCA on qihMT and T_1_w images yielded patterns that could best distinguish between RRMS and PMS. T_1_w-derived patterns discriminated PMS from RRMS with the highest sensitivity, whereas qihMT yielded the highest specificity. For a subset of 57% of patients with concordant results, a combination of qihMT and T_1_w could predict the PMS phenotype with an even higher degree of confidence.

Differentiating patterns in T_1_w, qMT, ihMTR, and qihMT reveal hyper-intense left temporal regions in PMS, while T_2_w and T_2_w-FLAIR signals are hypointense. This suggests less white matter in the temporal lobe of PMS compared to RRMS. The temporal lobe’s role in memory aligns with the observed memory impairment in PMS [26,27,28]. MT-based MRI methods, considered myelin-sensitive, showcase high-intensity voxels in PMS brains, indicating relatively preserved myelin density, while hypointense voxels suggest regions with myelin loss. The prevalence of hypointense voxels in the posterior fossa indicates degenerative/demyelinating processes in PMS, consistent with the motion-related neurological symptoms observed in PMS compared to RRMS [28,29].

SSM/PCA was able to differentiate MS phenotypes across most MRI methods, with qihMT and T_1_w exhibiting the highest AUC in ROC analysis. Notably, qihMT displayed the highest PPV and specificity, while T_1_w demonstrated the highest NPV and sensitivity. SSM/PCA on qihMT has a high certainty (87%) for the determination of PMS, while T_1_w reliably (93%) identified RRMS. Combining SSM/PCA on T_1_w and qihMT images enhances reliability, with only a 5% chance of misclassification. In cases of discordant results, SSM/PCA on qihMT may be preferred, given its higher PPV and significance in capturing RRMS to PMS transitions, influencing treatment strategies.

SSM/PCA applications in studies on dementias and Parkinsonian syndrome reported higher AUC, sensitivity, and specificity with larger PET datasets [23,30,31]. PET data’s quantitative nature and standardised reproducibility enhance its performance. MRI, being more variable, requires larger sample sizes for disease discrimination. Only one prior MRI study with SSM/PCA exists, combining PET and arterial spin labelling MRI, lacking sensitivity and specificity reporting, hampering comparisons with a study design more comparable to ours [31]. Despite MRI’s technical limitations and our modest sample size, our study demonstrates SSM/PCA’s proof-of-concept, achieving high sensitivity and specificity in RRMS and PMS patient identification.

Another study employing machine learning to differentiate MS phenotypes focussed on individuals with radiologically or clinically isolated syndromes, who were at risk of converting to MS [32]. In that study, ROC analysis using fractional anisotropy (FA) imaging yielded AUC values (0.71 to 0.78) similar to our study’s values, despite differences in patient categories, MRI sequences, and analysis methods. Another study aimed to distinguish RRMS from healthy controls, not among MS phenotypes, utilising T_1_w and myelin water fraction (MWF) [33]. Their ROC analysis reported AUC values of 0.67–0.83 for T_1_w, 0.72–0.84 for MWF, and 0.67–0.88 for their combination. Our direct comparison of RRMS vs. PMS (assumed to be more similar than healthy controls to MS patients) achieved an AUC of 0.70, highlighting SSM/PCA’s potential for detecting subtle differences between MS subtypes. While we explored post hoc whether concordance between T_1_w and qihMT results improved segregation, Yoo et al. employed specific algorithms during SSM/PCA, showing promising results [33]. This would be an interesting approach to further explore.

Heterogeneous disease presentation and random lesion distribution in MS pose challenges for differentiating methods. While lesions vary widely in location and size among MS patients, SSM/PCA robustly generated differentiating patterns, revealing shared pathological features [34] beyond periventricular lesion locations [35]. Despite phenotypic and interindividual variability, the technique’s ability to identify specific patterns using routine MRI sequences supports its proof of concept in MS research and care centres, requiring only approximately 7 min of extra scan time. Evaluation with a larger patient set can enhance the pattern robustness and phenotyping reliability. While PET studies showed SSM/PCA sensitivity to confounding factors, which are unexplored in MRI, they would likely influence the differentiating patterns. So, with robust patterns defined, the implementation of SSM/PCA would require scan normalisation, followed by subtraction of the GMP, and then comparison with the differentiating pattern. This would directly give a score regarding the probability of an MS phenotype.

## 5. Conclusions

This proof-of-concept study aimed to assess whether more information can be extracted from MRI scans that would allow differentiation of patients with different phenotypes of MS using SSM/PCA. Our proof-of-concept study indicates that SSM/PCA is able to differentiate between MS types using MRI. For differentiation between MS types, SSM/PCA on qihMT has a high specificity and positive predictive value, whereas SSM/PCA on T_1_w has a high sensitivity and high negative predictive value. Because the transition from RRMS to PMS is of particular interest, as it affects the treatment strategy, SSM/PCA on qihMT seems to be the preferred option for the accurate identification of PMS patients, because of its high specificity and PPV. When a patient can reliably be diagnosed with PMS, traditional DMTs are usually less effective and can better be substituted with an alternative treatment option, such as Ocrelizumab or Spinonimod. On the other hand, T_1_w is already integrated in standard clinical MRI protocol, and with its high sensitivity and NPP, T_1_w seems to have the best ability to identify RRMS patients. When qihMT and T_1_w predict the same phenotype—as is the case in the majority of patients—the MS phenotype can be predicted with very high reliability. Larger sample sizes and standardisation of MRI might also improve the results and enable SSM/PCA to produce more reliable disease-specific patterns.

## Figures and Tables

**Table 1:** Characteristics of the participants. Age, education, EDSS, number of lesions, and lesion volume are displayed as median values and range.

	RRMS	PMS
Number of participants	30	20
Gender (%male)	30	45
Age (y)	37 (19–49)	51 (33–62)
Education (y)	13 (6–20)	11 (5–20)
EDSS	2.5 (1–6)	6.5 (3.5–7.5)
Number of lesions	15 (1–37)	17 (2–32)
Lesion volume (mL)	10.3 (0.4–77.6)	10.6 (0.1–91.7)

**Table 2:** Validation of RRMS vs. PMS differentiating patterns using 1000 bootstraps followed by a leave-one-out cross-validation (LOOCV).

Image	Success%	LOOCV
T_1_w	85%	Yes
T_2_w	96%	Yes
T_2_w-FLAIR	93%	Yes
MTR	96%	No
qMT	94%	Yes
ihMTR	74%	Yes
qihMT	77%	Yes

**Table 3:** ROC analysis of the leave-one-out cross-validated differentiating patterns.

	AUC	Specificity	Sensitivity	NPV	PPV
T_1_w	0.68	60% 53%	87% 93%	82% 89%	68% 67%
T_2_w	0.58	60%	67%	75%	71%
T_2_w-FLAIR	0.46	87%	27%	58%	88%
MTR	-	-	-	-	-
qMT	0.61	80%	47%	83%	65%
		60%	67%	75%	71%
		47%	80%	65%	83%
ihMTR	0.58	53%	87%	88%	68%
qihMT	0.70	87%	67%	72%	83%

## Data Availability

Due to privacy regulations the clinical data collected in this study are not deposited in a public registry, but the data can be made available via a request to the corresponding author. The code for performing SSM/PCA is written in MATLAB and can also be made available upon request to the corresponding author.
